# Next-generation sequencing and a novel *COL3A1* mutation associated with vascular Ehlers–Danlos syndrome with severe intestinal involvement: a case report

**DOI:** 10.1186/s13256-016-1087-0

**Published:** 2016-10-31

**Authors:** Francesca Cortini, Barbara Marinelli, Manuela Seia, Barbara De Giorgio, Angela Cecilia Pesatori, Nicola Montano, Alessandra Bassotti

**Affiliations:** 1 Department of Clinical Sciences and Community Health, University of Milan IRCCS Ca’ Granda Foundation, Via San Barnaba 8, 20122 Milano, Italy; 2 Genetics Laboratory, IRCCS Ca’ Granda Foundation, via Francesco Sforza 35, Milan, Italy; 3 Department of Internal Medicine, Fondazione IRCCS Ca` Granda - Ospedale Maggiore & Department of Clinical Sciences and Health Community, University of Milan, Italy; 4 Regional Center of Ehlers-Danlos Syndrome, IRCCS Ca’ Granda Foundation, via San Barnaba 8, Milan, Italy

**Keywords:** Ehlers–Danlos syndrome vascular type, *COL3A1* gene, Next-generation sequencing, HaloPlex Target Enrichment, Sanger sequencing

## Abstract

**Background:**

The vascular type of Ehlers–Danlos syndrome is an autosomal dominant connective tissue disorder caused by a mutation in the *COL3A1* gene encoding pro-alpha1 chain of type III collagen. The vascular type of Ehlers–Danlos syndrome causes severe fragility of connective tissues with arterial and intestinal ruptures and complications in surgical and radiological treatments.

**Case presentation:**

We present a case of a 38-year-old Italian woman who was diagnosed as having the vascular type of Ehlers–Danlos syndrome. Genetic testing, conducted by Target Enrichment approach (Agilent Technologies), identified a new mutation c.1493G>A, p.G498D in exon 21 of *COL3A1* gene (heterozygous state). This mutation disrupts the normal glycine-X-Y repetitions of type III procollagen by converting glycine to aspartic acid.

**Conclusions:**

We report a new genetic mutation associated with the vascular type of Ehlers–Danlos syndrome. We also describe clinical and genetic findings that are important to understand the genotype/phenotype correlation in patients with the vascular type of Ehlers–Danlos syndrome.

## Background

Ehlers–Danlos syndrome (EDS) is a group of heterogeneous connective tissue disorders. It is characterized by abnormalities of the molecules that configure the extracellular matrix, such as collagen or its modifying enzymes [1]. The clinical characteristics of EDS are: hyperextensibility of the skin, hypermobility of the large joints, and easy bruising. On the basis of clinical experience and genetic findings, EDS is divided in six subtypes, (Villefranche classification 1997): (1) classic, which is the most frequent form; (2) hypermobility; (3) kyphoscoliosis; (4) arthrochalasia; (5) dermatosparaxis; and (6) vascular, which is the most dramatic form [2]. Genetic and clinical findings of EDS subtypes point out the genetic heterogeneity of EDS syndrome. Therefore, diagnosing the correct EDS type has important implications for genetic counseling and management. Any subtype of EDS is supported by specific biochemical and molecular investigations [1].

Vascular type of EDS (vEDS), also known as EDS type IV (NIM#130050), is a rare inherited autosomal dominant disorder with an estimated prevalence of 1 in 150,000. There are four main characteristics of vascular EDS: (1) rupture of blood vessels or internal organs such as the uterus and intestines, (2) an unusual facial appearance, (3) easy bruising, and (4) translucent skin with visible veins [3]. An important clinical event in vEDS is that the systemic arteries, which are rich in type III collagen, may undergo dissection, aneurysm, or rupture. These dramatic events may also occur spontaneously [4].

**Fig. 1:**
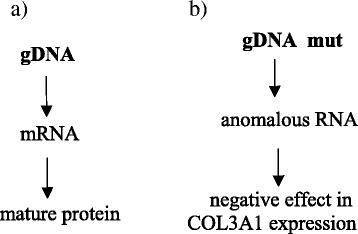
Schematic representation of mature type III collagen fibers in: **a** wild type and **b** mutant case respectively. If there are mutations in one *COL3A1* allele the dominant negative effect is a dramatic reduction in qualitative and quantitative production of COL3A1 protein. *gDNA* genomic DNA, *mRNA* messenger RNA, *mut* mutant

The genetic cause of vEDS is the presence of mutations in the collagen type III, alpha 1 gene (*COL3A1*), resulting in qualitative and/or quantitative abnormalities of mature type III collagen protein. Type III procollagen protein consists of 343 glycine (Gly)-X-Y repetitions (X and Y, any other amino acids) in each of the three amino acid chains. Mature type III collagen fiber comprises 3 alpha-1(III) chains. More than 200 mutations of the *COL3A1* gene have been described so far (www.le.ac.uk/ge/collagen), all of which lead to synthesis of an abnormal type III collagen protein (Fig. 1).

In this case report we describe a novel *COL3A1* point mutation (c.1493G>A, p.G498D) in a 38-year-old Italian woman. The diagnosis of vEDS was achieved at the Regional Center of Ehlers-Danlos syndrome (Ospedale Maggiore Policlinico, Milan, Italy); in addition to typical clinical characteristics of vEDS she referred an intestinal ischemic perforation of the sigmoid-rectum. The novel *COL3A1* mutation c.1493G>A, p.G498D that we describe here for the first time, is associated with profound change in the protein structure, causing vEDS.

## Case presentation

A 38-year-old Italian woman presented to our Regional Center of Ehlers-Danlos Syndrome, Ospedale Maggiore Policlinico, Milan, Italy, with a previous diagnosis of vEDS. She referred an intestinal ischemic perforation of the sigmoid-rectum with stercoral peritonitis treated with resection of her sigmoid-rectum and creation of a stoma. She did not have familial history of vEDS. When she was 26-years old and had not yet had a clinical diagnosis of EDS she underwent an orthopedic intervention for alignment of the internal arch plus tarsal sinus arthrodesis of both feet.

We applied next-generation sequencing (NGS) to investigate genomic regions of interest through target enrichment; it was performed by means of a HaloPlex Target Enrichment kit (Agilent Technologies). Probes for all coding exons, including intron–exon boundaries, were designed by the HaloPlex SureDesign website (www.genomics.agilent.com). The nine genes included in the design were selected on the basis of their clinical characteristics (www.le.ac.uk/ge/collagen; Table 1). The total region size was 100.336 kbp for an actual analyzed target of 99.718 kbp bases. Enrichment was performed according to the supplier’s protocol (version D.6, August 2014). A total of 225 ng DNA was digested in eight different restriction reactions during 30 minutes at 37 °C. The eight digestion reactions were combined into a single hybridization mix containing target-specific probes. Hybridization reaction was performed in a 3-hour reaction at 54 °C. Probes were labeled with biotin and designed to hybridize to both ends of the digested fragments, therefore generating circular fragments containing one nick. Then, the DNA probe hybrids were captured with streptavidin beads to eliminate linear non-target DNA fragments. In a second ligation reaction, the remaining nick was closed to complete circularization. The captured DNA was eluted from the beads and amplified by polymerase chain reaction (PCR) reaction, followed by a final purification reaction with AMPure XP beads (Beckman Coulter, Fullerton, CA, USA).

**Table 1:** List of genes included in the HaloPlex Target Enrichment panel (next-generation gene panel)

Gene	OMIM	Phenotype	Chromosome
*ADAMTS2*	604539	EDS type VIIC	chr5q.35.3
*B4GALT7*	604327	EDS progeroid type 1	chr5q35.3
*CHST14*	608429	EDS muscolocontractural type 1	chr15q15.1
*COL3A1*	120180	EDS type IV	chr2q32.2
*COL5A1*	120215	EDS classic type	chr9q34.3
*COL5A2*	120190	EDS classic type	chr2q32.2
*PLOD1*	153454	EDS type VI	chr1p36.22
*PLOD3*	603066	Lysyl hydroxylase 3 deficiency	chr7q22.1
*TNXB*	600985	EDS due to tenascin X deficiency	chr6p21.33

*EDS* Ehlers–Danlos syndrome, *OMIM* Online Mendelian Inheritance in Man

Finally, the concentration of each library was measured by TapeStation software and were diluted at 4nmol with TE. The final pool was obtained by mixing different genomic libraries and the ideal final concentration was 8 pM (ideal concentration for cluster density approximately 900/1000 K/mm^2^). Captured genomic libraries were sequenced to generate 2×150 bp paired end reads using the Illumina MiSeq.

To understand the generated FASTQ files (a file that stores a biological sequence and its quality score), a workflow was set up by Galaxy software (https://usegalaxy.org/) [5]. FASTQ files were evaluated by quality and they were trimmed, aligned, and mapped to the human reference genome (GRCh38/hg38) using Burrows-Wheeler Aligner [6, 7]. Alignments including sorting, merging, indexing, and generating alignments in a per-position format were manipulated by SAM tools [8, 9] to generate a BAM (binary format for storing sequence) file. Local realignment, base quality recalibration, and variant calling were performed using Genome Analysis Toolkit (GATK) version 2.0 (Broad Institute, Cambridge, MA, USA) [10] applying these quality parameters: coverage>20, base quality score>30, and mapping quality>20. Variants that did not pass these quality values were removed.

Finally, the Variant Calling Files (VCF) were filtered through different parameters such as genotype quality filters, gene feature filters (missense, splicing, frame shift), and functional impact filters using KGGSeq software [11]. The variants found with NGS target enrichment method were confirmed by Sanger method using BigDye Terminator Cycle Sequencing Kit (Applied Biosystems, Life Technologies) followed by capillary electrophoresis on an Applied Biosystems 3130xl (Applied Biosystems). Electropherograms were analyzed by Sequencher software v5.1.

To investigate the pathogenetic properties of a missense variant, different types of software were used: SIFT, PROVEAN, PolyPhen-2, Mutation Taster, Grantham distance, and Align GVGD; these types of software are based on sequence homology, the physiochemical similarity between the alternate amino acids, effect of an amino acid substitution on the structure and function of a protein, and conservation level of the amino acid residue among species [12–15].

Resequencing by target enrichment approach (HaloPlex technique) conducted using our custom panel (Table 1) showed the presence of an undescribed mutation in exon 21 of *COL3A1* gene at position 1493 leading to a nucleotide replacement (c.1493G>A, p.G498D) (www.le.ac.uk/ge/collagen/). It was confirmed by Sanger method on a second DNA extraction. Moreover, a complementary DNA (cDNA) analysis extracted from fibroblasts culture was not feasible due to the extreme fragility of the clinical state of our patient. 343 Gly-X-Y repetitions (X and Y, any other amino acids)

**Fig. 2:**
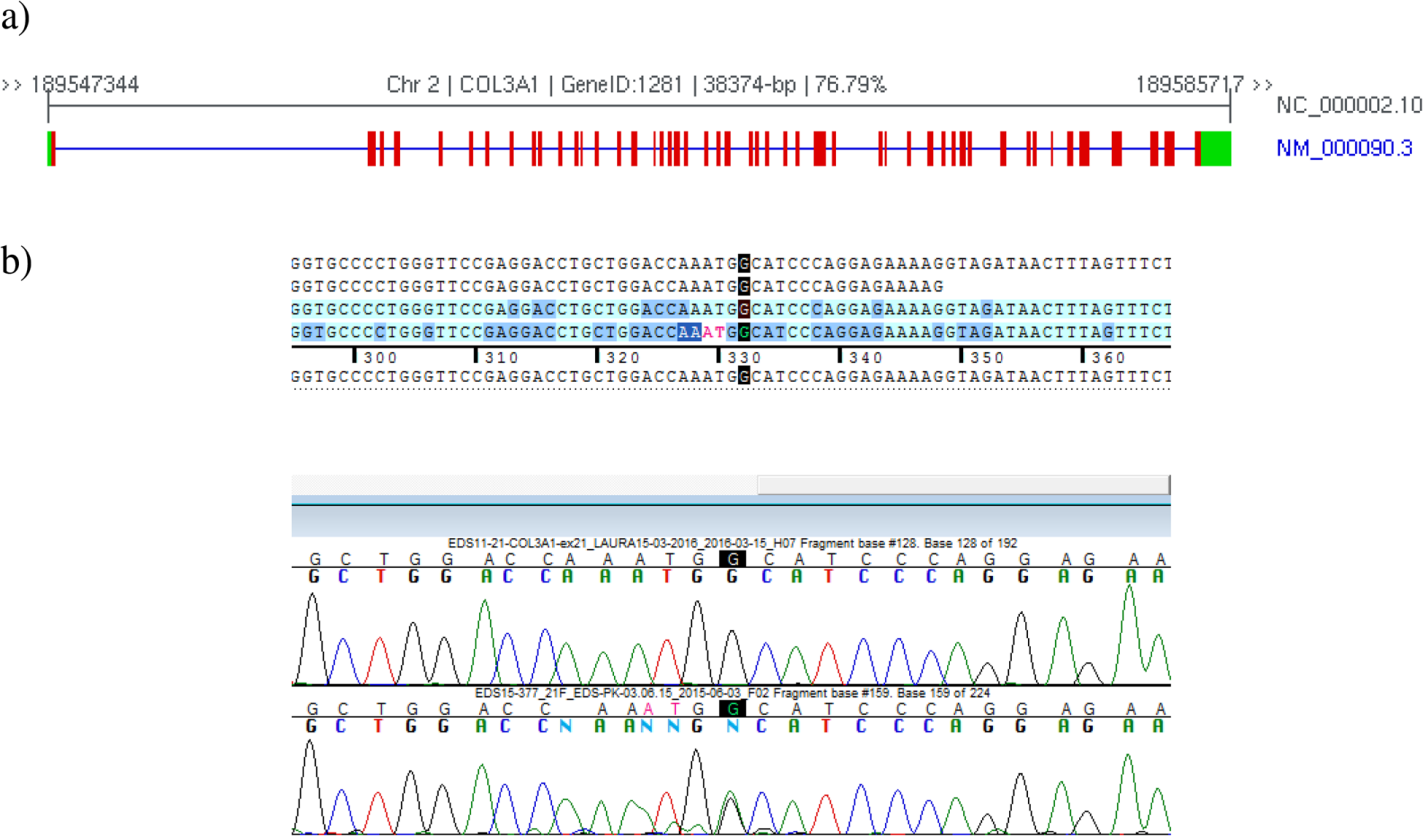
**a** Schematic structure of *COL3A1* gene (NG_007404.1) is located on chr2q31 and composed of 51 exons. **b** *COL3A1* DNA sequence in an unaffected individual and in a patient with vascular type of Ehlers–Danlos syndrome. Each nucleotide is indicated with a different color: *A* green, *C* blue, *G* black, *T* red

Figure 2 shows that c.1493G>A, p.G498D was a missense variant causing a substitution from Gly, a neutral amino acid, to aspartic acid, a polar amino acid, and it was located on the triple-helical region of the structure of the protein. Type III procollagen protein consists of 343 repetitions of Gly-X-Y (X and Y, any other amino acids) in each of three amino acids, which is a feature of collagen specificity. The mature type III collagen fibers consist of 3 alpha-1(III) chains. The mutation in one allele of *COL3A1* resulted in qualitative or quantitative abnormalities of mature type III collagen protein (Fig. 2) [16]. To evaluate the functional effect of mutation, we performed an *in silico* analysis using SIFT, PROVEAN, PolyPhen-2, and Align GVGD; all of these tools predicted that this change is causing the disease [12–15].

**Fig. 3:**
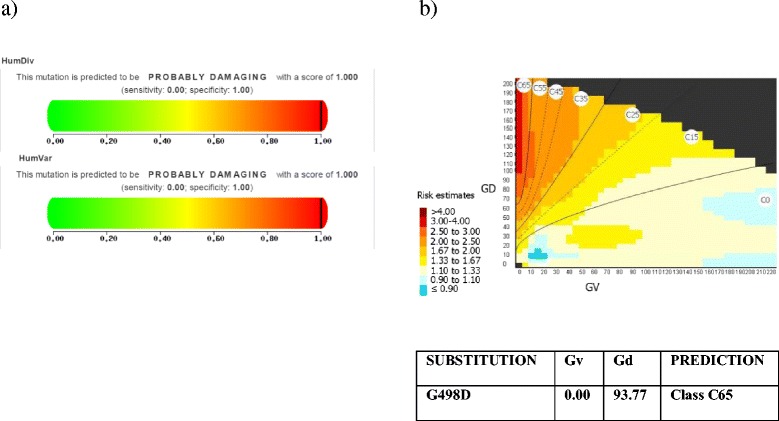
Data analysis. PolyPhen-2 prediction indicated that **a** c.1493G>A (p.G498D) probably damages the structure of the protein (on the basis of two different datasets Human Diversity and Human Variation). **b** Align GVGD indicated that c.1493G>A (p.G498D) is deleterious (class 65)

PolyPhen-2 software prediction, based on two datasets (Human Diversity and Human Variation), indicated that c.1493G>A, p.G498D is probably damaging for the structure of the protein with a score of 1.000 (sensitivity 0.00; specificity 1.00; Fig. 3). In particular, the Gly at 1493 position was extremely conserved through different species. SIFT and PROVEAN software, which evaluate whether an amino acid substitution has an impact on the biological function of the protein, confirmed the pathogenicity of the missense variant. Align GVGD, relying on the biophysical characteristics of the amino acids, predicted that this variant was not tolerated C65 (GV 0.00; GD 93.77; Fig. 3). It was impossible to analyze the effect of the mutation on the three-dimensional protein structure because at present there is no sequence homology reference to build the structure.

## Discussion

In the current case report we described a case of a 38-year-old Italian woman with typical symptoms of vEDS. She referred an ischemic perforation of her sigmoid-rectum with stercoral peritonitis treated with resection of sigmoid-rectum and creation of a stoma. The stoma was not closed to avoid surgical complications. Her intestinal problems worsened her clinical state. A physical examination revealed that her skin was slightly smooth and translucent with visible veins. Genetic analysis did not confirm any familial history.

Using a target NGS approach (HaloPlex protocol), a pathogenetic mutation c.1493G>A, p.G498D was discovered in the *COL3A1* gene (www.le.ac.uk/ge/collagen/). Up to now, more than 200 mutations in the *COL3A1* gene have been identified [17], all of which lead to the synthesis of an abnormal type III procollagen protein (Fig. 1). Approximately, two thirds of the mutations are single nucleotide substitutions that result in change for Gly residues in the triple helical domain of the proα1(III) chain. Most of the rest are splice site mutations that result in exon skipping, although some have more complex outcomes and a smaller number are larger genomic deletions [17]. There are no correlations between the nature or location of the mutation and the type or frequency of major complications [17].

The mutation c.1493G>A was detected in heterozygosity in the *COL3A1* gene, which resulted in a substitution of a Gly, a neutral amino acid, to aspartic acid, a polar amino acid. The meta-analysis, conducted by PolyPhen-2, SIFT, PROVEAN, and Align GVGD [12–15] showed that this point mutation was pathogenetic and was the cause of vEDS phenotype. The Gly substitution negatively influences the normal assembly of COL3A1 collagen fibers. In fact, the collagen characteristic is represented by the 343 repetitions of Gly-X-Y (X and Y, any other amino acids) [16]. The Gly amino acid is very important because it is the smallest amino acid and determines the link between the three peptide chains.

The mutation c.1493G>A, p.G498D was located on the triple helical region and it was theoretically conceivable that this type of mutation may change the characteristic triple helical structure and generate a severer phenotype. In fact, substitution of Gly in this region of the protein may strongly interfere with helix formation, delay the triple helix assembly, and lead to a severe modification. The negative effect was a dramatic decrease in the quantity and quality of COL3A1 protein. We did not have an opportunity to study the effect of this mutation on the three-dimensional protein structure because, in the literature, there is no sequence homology.

## Conclusions

We conclude that c.1493G>A, p.G498D identified in *COL3A1* gene was the cause of vEDS. The particular position of Gly substitution suggested a correlation between genotype and phenotype. The functional properties of c.1493G>A, p.G498D are still unknown, further studies on *in vitro* cell culture will be necessary to understand the link between this new variant and vEDS phenotype. The discovery of this new variant in vEDS underlines the importance of genetic tests as NGS in rare genetic diseases.
